# Clinical Text Summarization: Adapting Large Language Models Can Outperform Human Experts

**DOI:** 10.21203/rs.3.rs-3483777/v1

**Published:** 2023-10-30

**Authors:** Dave Van Veen, Cara Van Uden, Louis Blankemeier, Jean-Benoit Delbrouck, Asad Aali, Christian Bluethgen, Anuj Pareek, Malgorzata Polacin, Eduardo Pontes Reis, Anna Seehofnerová, Nidhi Rohatgi, Poonam Hosamani, William Collins, Neera Ahuja, Curtis P. Langlotz, Jason Hom, Sergios Gatidis, John Pauly, Akshay S. Chaudhari

**Affiliations:** 1 Department of Electrical Engineering, Stanford University, Stanford, CA, USA.; 2 Stanford Center for Artificial Intelligence in Medicine and Imaging, Palo Alto, CA, USA.; 3 Department of Computer Science, Stanford University, Stanford, CA, USA.; 4 Department of Electrical and Computer Engineering, The University of Texas at Austin, Austin, TX, USA.; 5 Department of Medicine, Stanford, CA, USA.; 6 University Hospital Zurich, Zurich, Switzerland.; 7 Copenhagen University Hospital, Copenhagen, Denmark.; 8 Albert Einstein Israelite Hospital, São Paulo, Brazil.; 9 Department of Radiology, Stanford University, Stanford, CA, USA.; 10 Department of Neurosurgery, Stanford University, Stanford, CA, USA.; 11 Department of Biomedical Data Science, Stanford, CA, USA.

## Abstract

Sifting through vast textual data and summarizing key information from electronic health records (EHR) imposes a substantial burden on how clinicians allocate their time. Although large language models (LLMs) have shown immense promise in natural language processing (NLP) tasks, their efficacy on a diverse range of clinical summarization tasks has not yet been rigorously demonstrated. In this work, we apply domain adaptation methods to eight LLMs, spanning six datasets and four distinct clinical summarization tasks: radiology reports, patient questions, progress notes, and doctor-patient dialogue. Our thorough quantitative assessment reveals trade-offs between models and adaptation methods in addition to instances where recent advances in LLMs may not improve results. Further, in a clinical reader study with ten physicians, we show that summaries from our best-adapted LLMs are preferable to human summaries in terms of completeness and correctness. Our ensuing qualitative analysis highlights challenges faced by both LLMs and human experts. Lastly, we correlate traditional quantitative NLP metrics with reader study scores to enhance our understanding of how these metrics align with physician preferences. Our research marks the first evidence of LLMs outperforming human experts in clinical text summarization across multiple tasks. This implies that integrating LLMs into clinical workflows could alleviate documentation burden, empowering clinicians to focus more on personalized patient care and the inherently human aspects of medicine.

## Introduction

Documentation plays an indispensable role in the practice of healthcare. Currently, clinicians spend significant time summarizing vast amounts of textual information—whether it be compiling diagnostic reports, writing progress notes, or synthesizing a patient’s treatment history across different specialists [3, 24, 30]. Even for experienced physicians with a high level of expertise, this intricate task naturally introduces the possibility for errors, which can be detrimental in a field where precision is paramount [6, 28, 76].

The widespread adoption of electronic health records (EHR) has expanded clinical documentation workload, directly contributing to increasing stress and clinician burnout [23, 29, 54]. Recent data indicates that physicians can expend two hours on documentation for each hour of patient interaction [60]. Meanwhile, documentation responsibilities for nurses consume up to 60% of their time and account for significant work stress [9, 22, 37]. These tasks divert attention from direct patient care, leading to worse outcomes for patients as well as disillusionment and decreased job satisfaction for clinicians [3, 55, 57, 68].

In recent years, large language models (LLMs) have gained remarkable traction, leading to widespread adoption of models such as ChatGPT [7], which excel at information retrieval, nuanced understanding, and text generation [8, 81]. While excellent LLM benchmarks for general NLP tasks exist [41, 82], they do not evaluate performance on relevant clinical tasks. Addressing this limitation presents a tremendous opportunity to accelerate the process of clinical text summarization, hence alleviating documentation burden and improving patient care.

Crucially, machine-generated summaries must be non-inferior to that of seasoned clinicians—especially when used to support sensitive clinical decision-making. Recent work in clinical natural language processing (NLP) has demonstrated potential on medical text [66, 75], adapting to the medical domain by either training a new model [59, 70], fine-tuning an existing model [67, 72], or supplying task-specific examples in the model prompt [46, 72]. However, adapting LLMs to summarize a diverse set of clinical tasks has not been thoroughly explored, nor has non-inferiority to humans been achieved.

With the overarching objective of bringing LLMs closer to clinical readiness, we aim to bridge the gap between theoretical promise and practical utility. We begin by implementing adaptation methods across eight open-source and proprietary LLMs for four distinct summarization tasks comprising six datasets. To our knowledge, the subsequent evaluation via NLP metrics is the most comprehensive assessment of contemporary LLMs for clinical text summarization. Our exploration illustrates the stark benefit of model adaptation over zero-shot prompting and delves into a myriad of trade-offs concerning different models and adaptation methods, revealing scenarios where advancements in model size, novelty, or domain specificity do not translate to superior performance.

Through a rigorous clinical reader study with ten physicians, we demonstrate that LLM summaries can surpass human summaries in terms of the following attributes: completeness, correctness, and conciseness. This novel finding affirms the non-inferiority of machine-generated summaries in a clinical context. We qualitatively analyze summaries to pinpoint challenges faced by both models and humans. Such insights can guide future enhancements of LLMs and their integration into clinical workflows. To support aligning future model outputs and NLP metrics directly to clinical preferences, we identify which metrics most correlate with reader scores on the aforementioned key attributes.

Our results demonstrate that LLMs often outperform human experts for clinical text summarization across the diverse range of documents we evaluate. This implies that LLMs could be leveraged to reduce documentation load and thus support clinicians—not supplant them. Once a summary is provided, clinicians are essential to make treatment recommendations and final decisions. Ultimately, such new tools may improve the clinical workflow [2], resulting in decreased clinician strain and improved patient care. Accelerating tedious tasks will enable healthcare providers to dedicate more time to the essential human facets of medicine, such as fostering patient relationships, understanding their specific goals, and offering personalized advice.

## Results

### Constructing prompt anatomy

**Figure 2 |:**
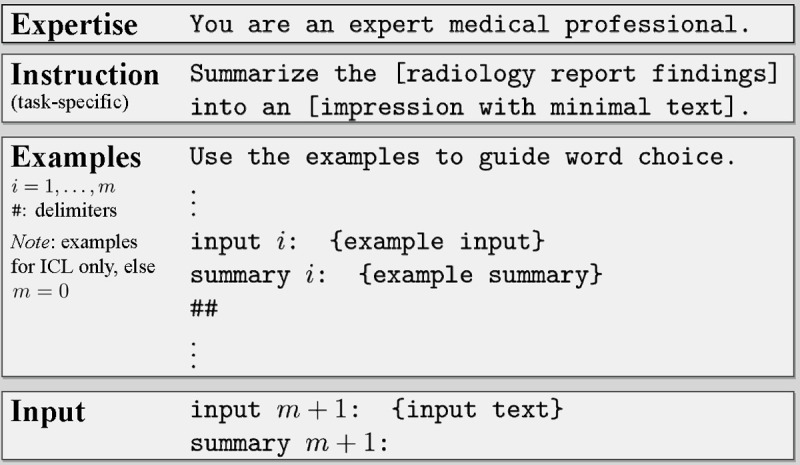
Prompt anatomy. Each summarization task uses a slightly different instruction, as depicted in Table A1.

We structured prompts (Figure 2) by following best practices [5, 56] and evaluating a handful of variants for each component. Table 1 demonstrates the effect of GPT-3.5 model expertise and temperature. For example, we achieved better performance by nudging the model to have expertise in medicine than expertise in wizardry, illustrating the value of this additional context for the target task. We also explored the temperature hyperparameter, which adjusts the LLM’s conditional probability distributions during sampling, hence affecting how often the model will output less likely tokens. Higher temperatures lead to more randomness and “creativity,” while lower temperatures produce more deterministic outputs. After searching over temperature values {0.1,0.5,0.9} using GPT-3.5, we found the lowest value, 0.1, performed best and thus set temperature to this value for all models. Intuitively, a lower value seems appropriate given our goal of factually summarizing text with a high aversion to hallucinations, or instances where the model generates factually incorrect text.

### Identifying the best model/method

**Figure 3 |:**
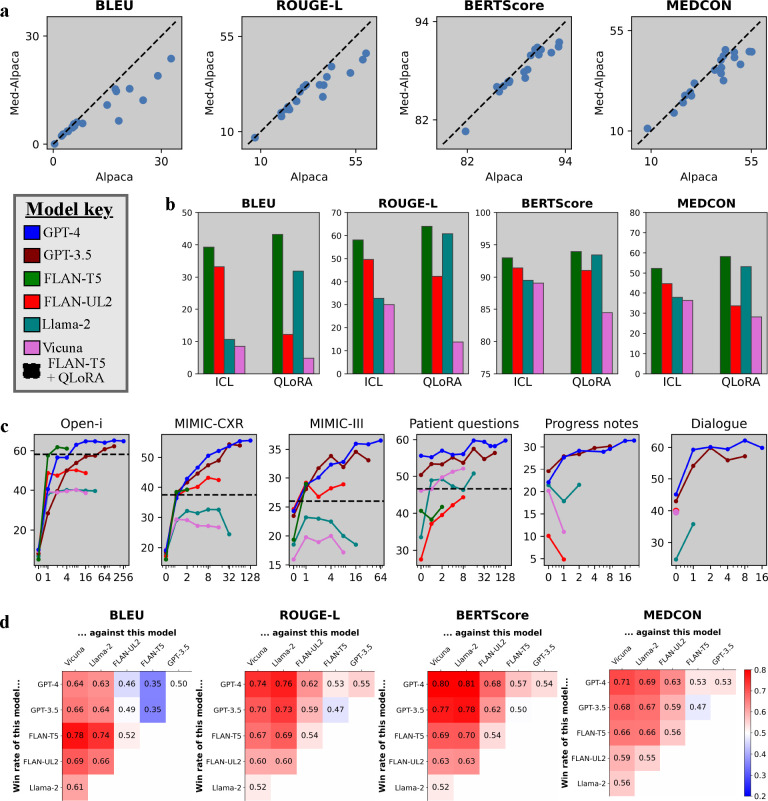
Quantitative results. **(a)** Alpaca vs. Med-Alpaca. Each data point corresponds to one experimental configuration, and the dashed lines denote equal performance. **(b)** One in-context example (ICL) vs. QLoRA methods across all open-source models on the Open-i radiology report dataset. **(c)** MEDCON scores vs. number of in-context examples across models and datasets. We also include the best model fine-tuned with QLoRA as a horizontal dashed line for valid datasets. See Figure A3 for results across all four metrics.**(d)** Model win rate: a head-to-head winning percentage of each model combination, where red/blue intensities highlight the degree to which models on the vertical axis outperform models on the horizontal axis.

When considering which open-source models to evaluate, we first assessed the benefit of fine-tuning open-source models on medical text. For example, Med-Alpaca [31] is a version of Alpaca [64] which was further instruction-tuned with medical Q&A text, consequently improving performance for the task of medical question-answering. Figure 3a compares these models for our setting, showing that most data points are below the dashed lines denoting equivalence. Hence despite Med-Alpaca’s adaptation for the medical *domain*, it actually performed worse than Alpaca for our tasks of clinical text summarization. This suggests that—in addition to domain adaptation–––task adaptation is also important. With this in mind, and considering that Alpaca is commonly known to perform worse than our other open-source autoregressive models Vicuna and Llama-2 [13, 82], for simplicity we excluded Alpaca and Med-Alpaca from further analysis.

Next, we compared ICL (in-context learning) vs. QLoRA (quantized low-rank adaptation) across the remaining open-source models using the Open-i radiology report dataset in Figure 3b and the patient health questions in Figure A2. We chose these datasets because their shorter context lengths allow for training with lower computational cost. FLAN-T5 generally performed best with QLoRA, although Llama-2 was often comparable. QLoRA typically outperformed ICL (one example) with the better models (FLAN-T5, Llama-2) but was often surpassed by ICL when more in-context examples were provided (Figure A3). Surprisingly, FLAN-T5 (2.7B) outperformed its fellow seq2seq model FLAN-UL2 (20B), despite being an older model with almost 10× fewer parameters.

Figure 3c displays MEDCON scores for all models against number of in-context examples up to the maximum number of examples permitted by each model and dataset. This graph also includes the best performing model (FLAN-T5) with QLoRA as a reference, depicted by a horizontal dashed line. Compared to zero-shot prompting (*m* = 0 examples), adapting with even *m* = 1 example delivered significantly improved performance in almost all cases, underscoring the importance of adaptation methods.While ICL and QLoRA were competitive for open-source models, proprietary models GPT-3.5 and GPT-4 far outperformed other models and methods given sufficient in-context examples. For a similar graph across all metrics, see Figure A3.

Figure 3d compares models using win rates, i.e. the head-to-head winning percentage of each model combination across the same set of samples. In other words, for what percentage of samples do model A’s summaries have a higher score than model B’s summaries? We deemed the best model and method to be GPT-4 (32K context length) with a maximum allowable number of in-context examples. We note that while FLAN-T5 was more competitive for syntactic metrics such as BLEU, this model is constrained to shorter context lengths (see Table 2).

### Analyzing reader study results

**Figure 4 |:**
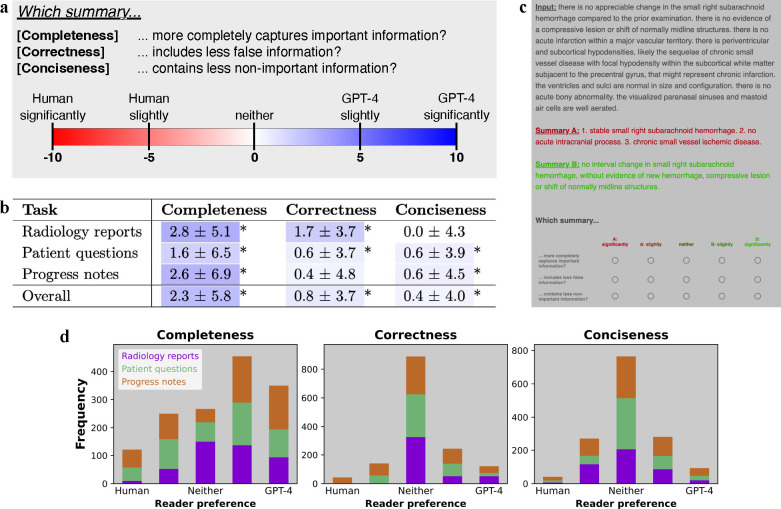
Clinical reader study. **(a)** Study design comparing the summarization of GPT-4 vs. that of human experts on three attributes: completeness, correctness, and conciseness. **(b)** Results. Highlight colors correspond to a value’s location on the color spectrum. Asterisks denote statistical significance by Wilcoxon signed-rank test, *p-value < 0.001. **(c)** Reader study user interface. **(d)** Distribution of reader scores for each summarization task across attributes. Horizontal axes denote reader preference as measured by a five-point Likert scale. Vertical axes denote frequency count, with 1,500 total reports for each plot.

**Figure 5 |:**
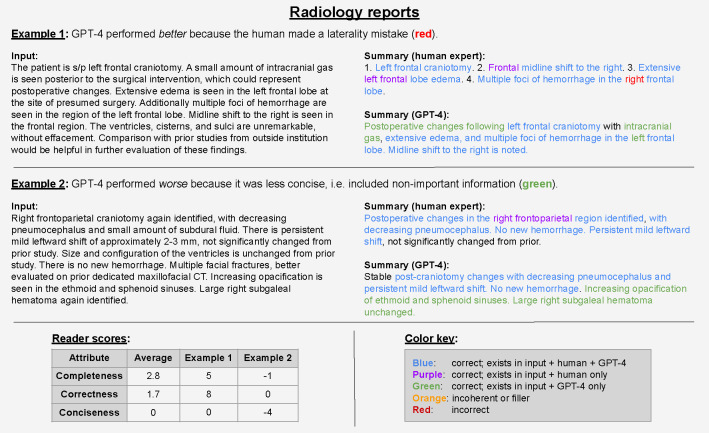
Annotation of two radiologist report examples from the reader study. The table (lower left) contains reader scores for these two examples and the task average across all samples.

Given our clinical reader study overview (Figure 4a), pooled results across our physicians (Figure 4b) demonstrate that GPT-4 summaries were more complete and concise fewer errors compared to human summaries. The distributions of reader responses in Figure 4c show that human summaries were preferred in only a minority of cases (19%), while in a majority GPT-4 was either non-inferior (45%) or preferred (36%). Table A2 contains scores separated by individual readers, while Table A3 affirms the reliability of scores across readers by displaying positive intra-reader correlation values. Based on physician feedback, we undertook a rigorous qualitative analysis to illustrate strengths and weaknesses of summaries by GPT-4 and humans; see Figures 5, A4, A5, and A6. Now, we discuss results with respect to each attribute individually.

We observed that GPT-4 summaries were more complete on average than human summaries, achieving statistical significance across all three summarization tasks with p-values < 0.001 (Figure 4b). We provide intuition for completeness by investigating a specific example in progress notes summarization. In Figure A5, GPT-4 correctly identified conditions that were missed by the human expert, such as “hypotension”, “anemia”, and “COPD”. GPT-4 was more *complete* in generating its progress note summary but also missed historical context (a history of “hypertension”, or “HTN”).

With regards to correctness, GPT-4 generated significantly fewer errors (p-value < 0.001) compared to human summaries (Figure 4b) overall and on two of three summarization tasks. For radiology reports, GPT-4 always matched or outperformed the human expert; across five readers’ comparisons of 100 samples, there were zero instances in which the human outperformed GPT-4 (Figure 4c). As an example of GPT-4’s superior correctness performance on the radiology report summarization task, we observe that it avoided common human errors related to lateral distinctions (right vs. left, Figure 5). For the problem list summarization task, Figure A5 demonstrates that GPT-4 avoided a mistake (including “UTI”) that was incorrectly documented by the human—for this example, the physician reader commented that “[the human] is hallucinating,” a phrase often used to describe mistakes made by LLMs. Despite this promising performance, GPT-4 was not perfect across all tasks. We see a clear example in Figure A6 where GPT-4 mistakenly generated (“hallucinated”) several conditions in the problem list that were false, such as “eosinophilia”.

With regards to conciseness, GPT-4 performed significantly better (p-value < 0.001) overall and on two of the three tasks. However, for radiology reports, the conciseness of GPT-4 was similar to that of human experts. See Figure 5 for an example in which GPT-4’s summary includes correct information which readers deemed non-important.

### Connecting quantitative and clinical evaluations

**Figure 6 |:**
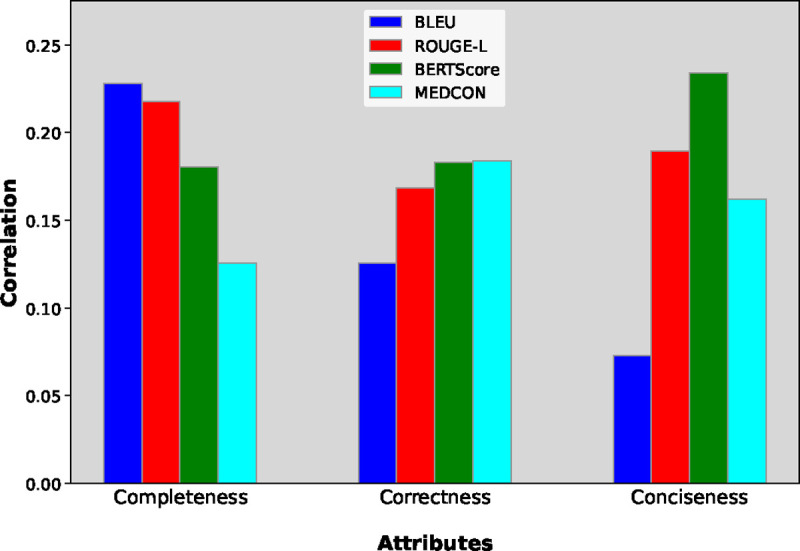
Spearman correlation coefficients between NLP metrics and reader preference assessing completeness, correctness, and conciseness.

We created Figure 6 to capture the correlation between NLP metrics and physicians’ preference. These values are calculated as the Spearman correlation coefficient between NLP metric scores and the magnitudes of reader scores. For correctness, the semantic metric BERTScore and conceptual metric MEDCON correlated most strongly with reader preference. Meanwhile, the syntactic BLEU metric correlated most with completeness and least with conciseness. Given that BLEU measures sequence overlap, this result seems reasonable, as more text provides more “surface area” for overlap and reduces the brevity penalty that BLEU applies on generated sequences which are shorter than the reference [51]. While these results demonstrate that some metrics are more useful for measuring particular attributes, the low magnitude of correlation values (approximately 0.2) underscores the need to go beyond NLP metrics when assessing clinical readiness.

## Discussion

In this research, we exhaustively evaluated methods for adapting LLMs to summarize clinical text, analyzing eight models across a diverse set of summarization tasks. Our quantitative results underscore the advantages of adapting models to specific tasks and domains. The ensuing clinical reader study demonstrates that LLM summaries are often preferred over human expert summaries due to higher scores for completeness, correctness, and conciseness. The subsequent qualitative exploration provides deeper insights into the limitations of both LLMs and human experts. Novel evidence from our research suggests a promising avenue for LLMs—not as replacements for clinicians, but as tools to reduce documentation burden and so that clinicians can direct more attention toward patient care. Now, we discuss insights and future steps enabled by this work.

We first highlight the importance of “prompt engineering,” or modifying and tuning the input prompt to improve model performance. This is well-reflected in our evaluation of conciseness. We specified the desired summary length in the instruction, for example with “one question of 15 words or less” for summarizing patient questions (Table A1). Without this instruction, the model might generate lengthy outputs—occasionally even longer than the input text. When considering conciseness scores (Figure 4b), radiology reports were the only task in which physicians did not prefer GPT-4’s summaries to the human experts. This could be attributed to the relatively vague length specification in the radiology reports instruction, i.e. “…with minimal text,” while the other two task instructions quantify length.

Overall, we achieve strong results while performing a basic search across 1–2 options for each task instruction (Table A1). Prompt phrasing and model temperature can be very important for a LLM, as demonstrated in the literature [62, 73] and in Table 1. This suggests better results could be achieved via further study of prompt engineering and model hyperparameters, which we leave for future work.

Model performance generally improved with more context. Even one example provided significant benefit compared to zero-shot prompting, hence underscoring the value of adaptation methods. Note that the number of allowable examples depends on the number of tokens per example and the model context length. This motivates future work to pursue more challenging tasks such as summarizing longer documents or multiple documents of different types. Addressing these cases demands two key advancements: (1) extending GPT-4’s current context length beyond 32,768 tokens, potentially through multi-query aggregation or methods which increase context length [21, 52], and (2) introducing open-source datasets that include broader tasks and lengthier documents.

Now, we discuss trade-offs between lightweight adaptation methods. While QLoRA fine-tuning performed comparably for some cases, ICL triumphed overall, particularly when including proprietary models GPT-3.5 and GPT-4. The proprietary nature of these models raises an interesting point for healthcare, where data and model governance are important—especially if summarization tools are cleared for clinical use by the FDA. This could motivate the use of fine-tuning methods on open-source models. Governance aside, ICL provides many benefits: (1) model weights are fixed, hence enabling queries of pre-existing LLMs (2) adaptation is feasible even a few examples, while fine-tuning methods such as QLoRA typically require hundreds or thousands of examples.

We consider trade-offs of different model types: autoregressive and sequence-to-sequence (seq2seq). Seq2seq models (FLAN-T5, FLAN-UL2) performed very well on syntactical metrics such as BLEU but worse on others (Figure 3d), suggesting that these models excel more at matching word choice than matching semantic or conceptual meaning. Note seq2seq models are often constrained to much shorter context length than autoregressive models such as GPT-4, because seq2seq models require the memory-intensive step of encoding the input sequence into a fixed-size context vector. Among open-source models, seq2seq (FLAN-T5, FLAN-UL2) performs better than autoregressive (Llama-2, Vicuna) models on radiology reports but worse on patient questions and progress notes (Figure 3c). Given that these latter datasets have higher lexical variance (Table 3) and more heterogeneous formatting compared to radiology reports, we posit that autoregressive models may perform better with increasing data heterogeneity and complexity.

The overwhelming evidence from our reader study suggests that adapting LLMs can outperform human experts in terms of completeness, correctness, and conciseness. When qualitatively analyzing summaries, we notice a few general trends. As implied by the completeness scores, GPT-4 excelled at identifying and understanding the most relevant information from the source text. However, both GPT-4 and human experts faced challenges interpreting ambiguity, such as user queries in patient health questions. Consider Example 1 of Figure A4, in which the input question mentioned “diabetes and neuropathy.” GPT-4 mirrored this phrasing verbatim, while the human expert interpreted it as “diabetic neuropathy.” This highlights GPT-4’s tendency toward a literal approach without interpretation, which may either be advantageous or limiting. In Example 2 of Figure A4, GPT-4 simply reformulated the input question about tests and their locations, while the human inferred a broader query about tests and treatments. In both cases, GPT-4’s summaries leaned toward literalness, a trait that readers sometimes favored and sometimes did not. In future work, a systematic exploration of model temperature could further illuminate this trade-off.

Model hallucinations—or instances of factually incorrect text—present a notable barrier to the clinical integration of LLMs, especially considering the high degree of accuracy required for medical applications. Our reader study results for correctness (Figure 4b) illustrate that hallucinations are made less frequently by our adapted LLMs than by humans. This implies that incorporating LLMs could actually reduce summarization errors in clinical practice. Beyond the scope of our work, there’s further potential to reduce hallucinations through incorporating checks by a human, checks by another LLM, or using a model ensemble to create a “committee of experts” [10, 36].

Now, we discuss general trends for our clinical NLP metrics. The syntactic metric BLEU provided the highest correlation with physician preference for completeness. Given that BLEU measures sequence overlap, this result seems reasonable, as more text provides more “surface area” for overlap; more text also reduces the brevity penalty that BLEU applies on generated sequences which are shorter than the reference [51]. Meanwhile the metrics BERTScore and MEDCON correlated most strongly with physician preference for correctness. This implies that the semantics (BERTScore) and concepts (MEDCON) measured by these metrics correspond to correctness more effectively than syntactic metrics BLEU and ROUGE-L.

Many clinical NLP papers rely primarily on quantitative metrics for evaluation. Given the critical nature of medical tasks, demonstrating clinical readiness requires including human experts in the evaluation process. To address this, there have been recent releases of expert evaluations for adjacent clinical NLP tasks [24, 79]. Other work employs human experts to evaluate synthesized abstracts, demonstrating that NLP metrics are not sufficient to measure summary quality [63]. Aside from the low correlation values in Figure 6, our reader study results in Figure 4 also highlight another limitation of NLP metrics, especially as model-generated summaries become increasingly viable. These metrics rely on a reference, which we have demonstrated can be fallible. Hence we advocate that human evaluation is essential when assessing the clinical feasibility of new methods. When human evaluation is not feasible, Figure 6 suggests that syntactic metrics are better at measuring completeness, while semantic and conceptual metrics are better at measuring correctness.

This study has several limitations which motivate further work. First, we do not consider the inherently context-specific nature of summarization. For example, a gastroenterologist, radiologist, and oncologist may have different preferences for summaries of a cancer patient with liver metastasis. Or perhaps an abdominal radiologist will want a different summary than a neuroradiologist. Further, individual clinicians may prefer different styles or amounts of information. While we do not explore such a granular level of adaptation, this may not require much further development: since our best results were obtained via ICL with a handful of examples, one could plausibly adapt using examples curated for a particular specialty or clinician. Another limitation is that radiology report human summaries occasionally recommend further studies or refer to prior studies, e.g. “... not significantly changed from prior” in Figure 5. These instances are out of scope for the LLM, as it does not have access to prior studies nor the purview to make recommendations. Hence for our clinical reader study, physicians were told to disregard these phrases. However in future work, it would be interesting to provide more context via prior reports and allow the LLM to make a treatment suggestion.

## Reproducibility

In an effort to disseminate these methods for further validation and clinical impact, we will make our code publicly available at github.com/StanfordMIMI/clin-summ prior to publication. While all datasets are publicly available, we will share our preprocessed versions for those which do not require Physionet [35] access: Open-i [19] (radiology reports), MeQSum [4] (patient questions), and ACI-Bench [78] (dialogue).

## Methods

### Large language models

We investigated a diverse collection of transformer-based LLMs for clinical summarization tasks. This included two broad approaches to language generation: sequence-to-sequence (seq2seq) models and autoregressive models. Seq2seq models use an encoder-decoder architecture to map the input text to a generated output, often requiring paired datasets for training. These models have shown strong performance in machine translation [11] and summarization [58]. In contrast, the autoregressive models typically only use a decoder. They generate tokens sequentially—where each new token is conditioned on previous tokens—thus efficiently capturing context and long-range dependencies. Autoregressive models are typically trained with unpaired data, and they are particularly useful for NLP tasks such as text generation, question-answering, and dialogue interactions [7, 13].

We included prominent seq2seq models due to their strong summarization performance [58] and autoregressive models due to their state-of-the-art performance across general NLP tasks [82]. As shown in Table 2, our choice of models varied widely with respect to number of parameters (2.7 billion to 175 billion) and context length (512 to 32,000), i.e. the maximum number of input tokens a model can process. We organized our models into three categories:

#### Open-source seq2seq models.

The original T5 “text-to-text transfer transformer” model [53] demonstrated excellent performance in transfer learning using the seq2seq architecture. A derivative model, FLAN-T5 [14, 43], improved performance via instruction prompt tuning. This T5 model family has proven effective for various clinical NLP tasks [40, 72]. The FLAN-UL2 model [15, 65] was introduced recently, which featured an increased context length (four-fold that of FLAN-T5) and a modified pre-training procedure called unified language learning (UL2).

#### Open-source autoregressive models.

The Llama family of LLMs [69] has enabled the proliferation of open-source instruction-tuned models that deliver comparable performance to GPT-3 [7] on many benchmarks despite their smaller sizes. Descendants of this original model have taken additional fine-tuning approaches, such as fine-tuning via instruction following (Alpaca [64]), medical Q&A data (Med-Alpaca [31]), user-shared conversations (Vicuna [13]), and reinforcement learning from human feedback (Llama-2 [69]). Llama-2 allows for two-fold longer context lengths (4,096) relative to the aforementioned open-source autoregressive models.

**Figure A1 |:**
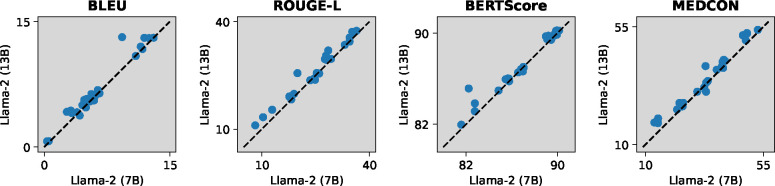
Comparing Llama-2 (7B) vs. Llama-2 (13B). The dashed line denotes equivalence, and each data point corresponds to the average score of *s* = 250 samples for a given experimental configuration, i.e. {dataset × *m* in-context examples}.

**Figure A2 |:**
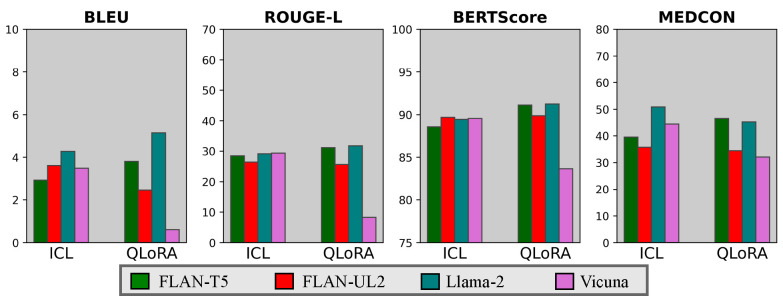
Summarization performance comparing one in-context example (ICL) vs. QLoRA across all open-source models on patient health questions. Figure 3b contains similar results with the Open-i radiology report dataset.

**Figure A3 |:**
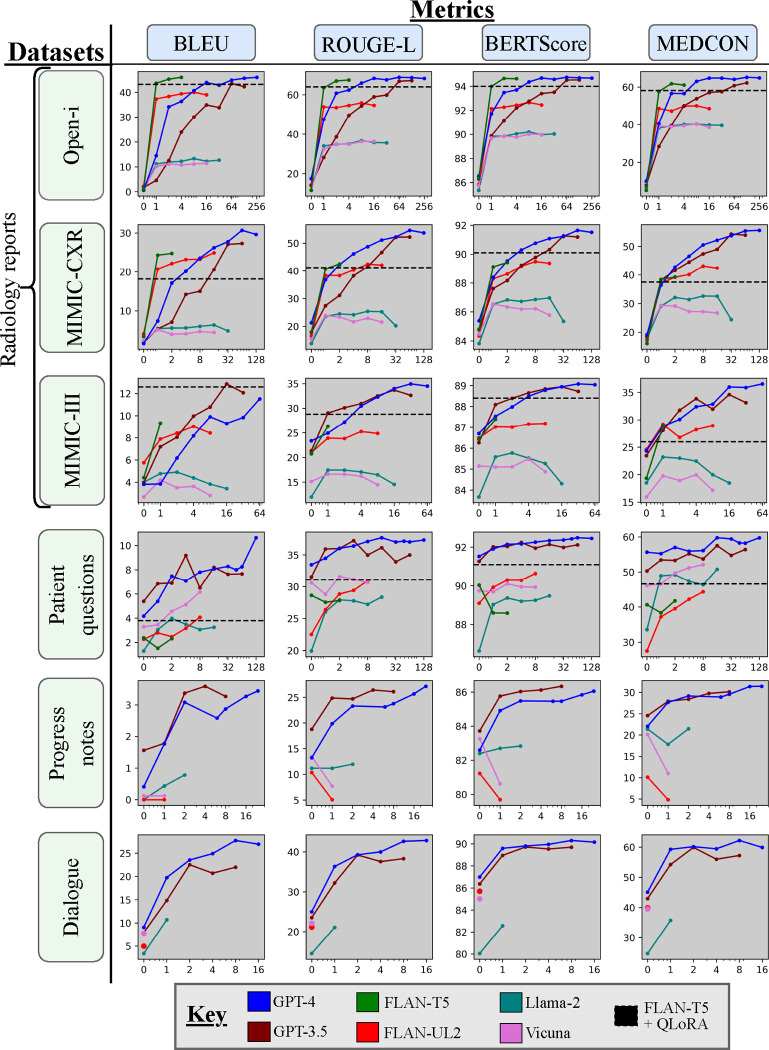
Metric scores vs. number of in-context examples across models and datasets. We also include the best model fine-tuned with QLoRA (FLAN-T5) as a horizontal dashed line.

Our focus was primarily on the 7B-parameter tier of these models, despite some models such as Llama-2 having larger versions. The benefit of larger models is explored in Figure A1, which found this improvement marginal for Llama-2 (13B) compared to Llama-2 (7B). While other open-source models might have slightly outperformed our selections, this likely wouldn’t have significantly changed our analysis—especially since the clinical reader study employed a state-of-the-art proprietary model [82].

#### Proprietary autoregressive models.

We include GPT-3.5 [49] and GPT-4 [50], the latter of which is widely regarded as state-of-the-art on general NLP tasks [82]. Both models offer significantly higher context length (16,384 and 32,768) than open-source models.

### Adaptation methods

We considered two proven techniques for adapting pre-trained general-purpose LLMs to domain-specific clinical summarization tasks:

#### In-context learning (ICL).

ICL is a lightweight adaptation method that requires no altering of model weights; instead, one includes a handful of in-context examples directly within the model prompt [39]. This simple approach provides the model with context, enhancing LLM performance for a particular task or domain [46, 72]. We implemented this by choosing, for each sample in our test set, the *m* nearest neighbors training samples in the embedding space of the PubMedBERT model [16]. Note that choosing “relevant” in-context examples has been shown to outperform choosing examples at random [47]. For a given model and dataset, we used *m* = 2^*x*^ examples, where *x* ∈{0,1,2,3*,…,M*} for *M* such that no more than 1% of the *s* = 250 samples were excluded due to prompts exceeding the model’s context length. Hence each model’s context length limited the allowable number of in-context examples.

To demonstrate the benefit of adaptation methods, we included the baseline zero-shot prompting, i.e. *m* = 0 in-context examples.

#### Quantized low-rank adaptation (QLoRA).

Low-rank adaptation (LoRA) [32] has emerged as an effective, lightweight approach for fine-tuning LLMs by altering a small subset of model weights—often < 0.1% [72]. LoRA inserts trainable matrices into the attention layers; then, using a training set of samples, this method performs gradient descent on the inserted matrices while keeping the original model weights frozen. Compared to training model weights from scratch, LoRA is much more efficient with respect to both computational requirements and the volume of training data required. Recently, QLoRA [20] has been introduced as a more memory-efficient variant of LoRA, employing 4-bit quantization to enable the fine-tuning of larger LLMs given the same hardware constraints. This quantization negligibly impacts performance [20]; as such, we use QLoRA for all model training. Note that QLoRA could not be used to fine-tune proprietary models on our consumer hardware, as their model weights are not publicly available.

### Data

**Figure A4 |:**
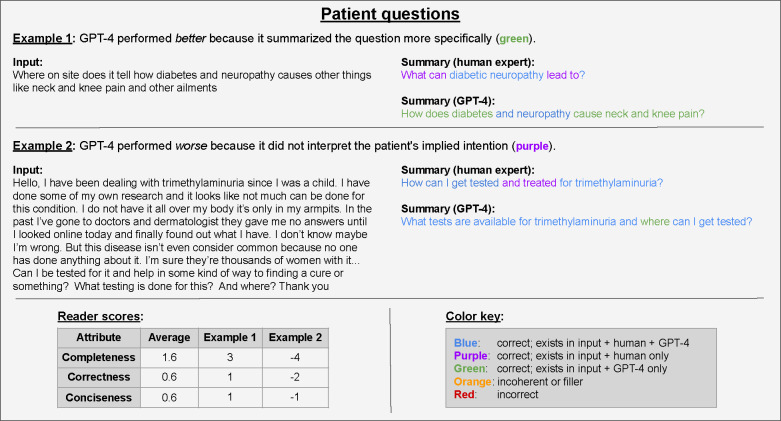
Annotation of two patient health question examples from the reader study. The table (lower left) contains reader scores for these two examples and the task average across all samples.

**Figure A5 |:**
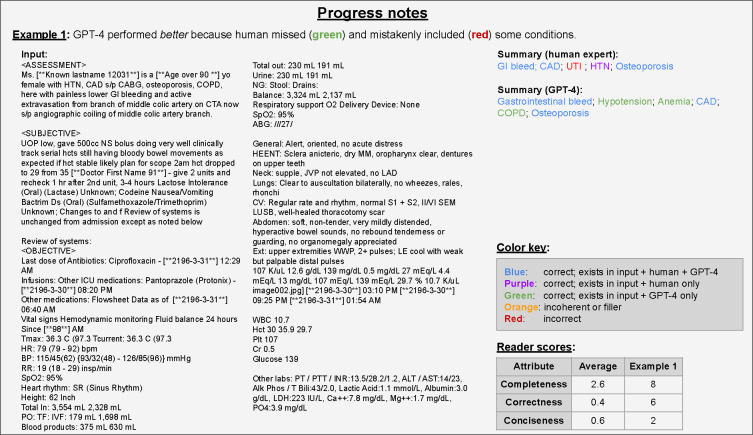
Annotation of a progress notes summarization example evaluated in the reader study. The table (lower right) contains reader scores for this example and the task average across all samples.

**Figure A6 |:**
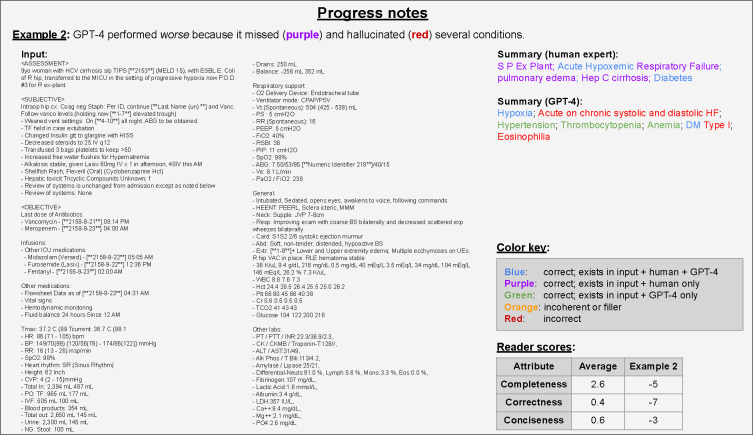
Annotation of a progress notes summarization example evaluated in the reader study. The table (lower right) contains reader scores for this example and the task average across all samples.

**Figure A7 |:**
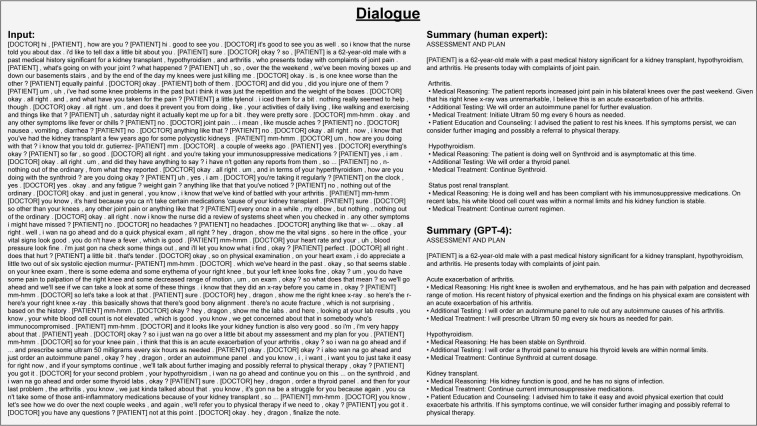
Example of the doctor-patient dialogue summarization task, including “assessment and plan” sections generated by both a human expert and GPT-4.

To robustly evaluate LLM performance on clinical text summarization, we chose four distinct summarization tasks, comprising six open-source datasets. As depicted in Table 3, each dataset contained a varying number of samples, token lengths, and lexical variance. Lexical variance is calculated as $\frac{\text{number}\;\text{of}\;\text{unique}\;\text{words}}{\text{number}\;\text{of}\;\text{total}\;\text{words}}$ across the entire dataset; hence a higher ratio indicates less repetition and more lexical diversity. We describe each task and dataset below. For examples of each task, please see Figures 5, A4, A5, A6, and A7.

#### Radiology reports

Radiology report summarization takes as input the findings section of a radiology study containing detailed exam analysis and results. The goal is to summarize these findings into an impression section, which concisely captures the most salient, actionable information from the study. We considered three datasets for this task, where both reports and findings were created by attending physicians as part of routine clinical care. Open-i [19] contains de-identified narrative chest x-ray reports from the Indiana Network for Patient Care 10 database. From the initial set of 4K studies, Demner-Fushman *et al.* [19] selected a final set of 3.4K reports based on the quality of imaging views and diagnostic content. MIMIC-CXR [33] contains chest x-ray studies accompanied by free-text radiology reports acquired at the Beth Israel Deaconess Medical Center between 2011 and 2016. For this study, we used a dataset of 128K reports [12] preprocessed by the RadSum23 shared task at BioNLP 2023 [17, 18]. MIMIC-III [34] contains 67K radiology reports spanning seven anatomies (head, abdomen, chest, spine, neck, sinus, and pelvis) and two modalities: magnetic resonance imaging (MRI) and computed tomography (CT). This dataset originated from patient stays in critical care units of the Beth Israel Deaconess Medical Center between 2001 and 2012. For this study, we utilized a preprocessed version via RadSum23 [17, 18]. Compared to x-rays, MRIs and CT scans capture more information at a higher resolution. This usually leads to longer reports (Table 3), rendering MIMIC-III a more challenging summarization dataset than Open-i or MIMIC-CXR.

#### Patient questions

Question summarization consists of generating a condensed question expressing the minimum information required to find correct answers to the original question [4]. For this task, we employed the MeQSum dataset [4]. MeQSum contains (1) patient health questions of varying verbosity and coherence selected from the U.S. National Library of Medicine (2) corresponding condensed questions created by three medical experts such that the summary allows retrieving complete, correct answers to the original question without the potential for further condensation. These condensed questions were then validated by two physicians and verified to have high inter-annotator agreement. Due to the wide variety of these questions, MeQSum exhibited the highest lexical variance of our datasets (Table 3).

#### Progress notes

The goal of this task is to generate a “problem list,” or condensed list of diagnoses and medical problems using the provider’s progress notes during hospitalization. For this task, we employed the ProbSum dataset [26]. This dataset was extracted from the MIMIC-III database of de-identified hospital intensive care unit (ICU) admissions. ProbSum contains (1) progress notes averaging > 1,000 tokens and substantial presence of unlabeled numerical data, e.g. dates and test results (2) corresponding problem lists created by attending medical experts in the ICU. We accessed this data via the BioNLP Problem List Summarization shared task [18, 26, 27] and Physionet [35].

#### Dialogue

The goal of this task is to summarize a doctor-patient conversation into an “assessment and plan” paragraph. For this task, we employed the ACI-Bench dataset [1, 77, 78], which contains (1) 207 doctor-patient conversations (2) corresponding patient visit notes, which were first generated by a seq2seq model and subsequently corrected and validated by expert medical scribes and physicians. Since ACI-Bench’s visit notes include a heterogeneous collection of section headers, we chose 126 samples containing an “assessment and plan” section for our analysis. Per Table 3, this task entailed the largest token count across our six datasets for both the input (dialogue) and target (assessment).

### Experimental Setup

For each dataset, we constructed test sets by randomly drawing the same s samples, where s=250 for all datasets except dialogue (s=100), which included only 126 samples in total. After selecting these s samples, we chose another s as a validation set for datasets which incorporated fine-tuning. We then used the remaining samples as a training set for ICL examples or QLoRA fine-tuning.

We leveraged PyTorch for our all our experiments, which included the parameter-efficient fine-tuning [45] and the generative pre-trained transformers quantization [25] libraries for implementing QLoRA. We fine-tuned models with QLoRA for five epochs using the Adam optimizer with weight decay fix [44]. An initial learning rate of $1e^{-3}$ was decayed linearly to $1e^{-4}$ after a 100-step warm-up; we determined this configuration after experimenting with different learning rates and schedulers. To achieve an effective batch size of 24 on each experiment, we adjusted both individual batch size and number of gradient accumulation steps to fit on a single consumer GPU, a NVIDIA Quadro RTX 8000. All open-source models are available on HuggingFace [74].

### Quantitative metrics

We used well-known summarization metrics to assess the quality of generated summaries. BLEU [51], the simplest metric, calculates the degree of overlap between the reference and generated texts by considering 1- to 4-gram sequences. ROUGE-L [42] evaluates similarity based on the longest common subsequence; it considers both precision and recall, hence being more comprehensive than BLEU. In addition to these syntactic metrics, we employed BERTScore, which leverages contextual BERT embeddings to evaluate the semantic similarity of the generated and reference texts [80]. Lastly, we included MEDCON [78] to gauge the consistency of medical concepts. This employs QuickUMLS [61], a tool that extracts biomedical concepts via string matching algorithms [48]. MEDCON was restricted to relvant UMLS semantic groups (Anatomy, Chemicals & Drugs, Device, Disorders, Genes & Molecular Sequences, Phenomena and Physiology). All four metrics ranged from [0,100] with higher scores indicating higher similarity between the generated and reference summaries.

### Reader study

After identifying the best model and method via NLP quantitative metrics, we performed a clinical reader study across three summarization tasks: radiology reports, patient questions, and progress notes. The dialogue task was excluded due to the unwieldiness of a human reader parsing many lengthy transcribed conversations and paragraphs; see Figure A7 for an example and Table 3 for the token count.

Our readers included two sets of physicians: (1) five board-certified radiologists to evaluate summaries of radiology reports (2) five board-certified hospitalists (internal medicine physicians) to evaluate summaries of patient questions and progress notes. For each task, each physician viewed the same 100 randomly selected inputs and their A/B comparisons (human vs. model summaries), which were presented in a blinded and randomized order. An ideal summary would contain all clinically significant information (*completeness*) without any errors (*correctness*) or superfluous information (*conciseness*). Hence we posed the following three questions for readers to evaluate using a five-point Likert scale.

- **Completeness:** “Which summary more completely captures important information?” This compares the summaries’ recall, i.e. the amount of clinically significant detail retained from the input text.
- **Correctness:** “Which summary includes less false information?” This compares the summaries’ precision, i.e. instances of false information due to hallucination by the model or an error by the human expert.
- **Conciseness:** “Which summary contains less non-important information?” This compares which summary is more condensed, as the value of a summary decreases with superfluous information.

Figure 4c demonstrates the user interface for this study, which we created and deployed via Qualtrics.

Given this non-parametric, categorical data, we assessed the statistical significance of responses using a Wilcoxon signed-rank test with Type 1 error rate = 0.05, adjusted for multiple comparisons using the Bonferroni correction. We estimated intra-reader correlation based on a mean-rating, fixed agreement, two-may mixed effects model [38] using the Pingouin package [71]. Additionally, readers provided comments on notable samples to identify interesting observations for qualitative analysis.

To obfuscate any formatting differences between the human and model summaries, we applied simple post-processing to standardize capitalization, punctuation, newline characters, etc.

### Connecting quantitative and clinical evaluations

We now outline our calculation of correlation values between NLP metrics and clinical reader scores in Figure 6. Note that in our work, these tools measured different quantities: NLP metrics measured the similarity between two summaries, while reader scores measured which summary is better. Consider an example where two summaries are exactly the same: NLP metrics would yield the highest possible score (100), while clinical readers would provide a score of 0 to denote equivalence. As the magnitude of a reader score increases, the two summaries are increasingly dissimilar, yielding a lower quantitative metric score. Hence, the correlation values are calculated as the Spearman correlation coefficients between NLP metric scores and the magnitudes of the reader scores. Since these features are inversely correlated, for clarity we display the negative correlation coefficient values.

## Figures and Tables

**Figure 1 |:**
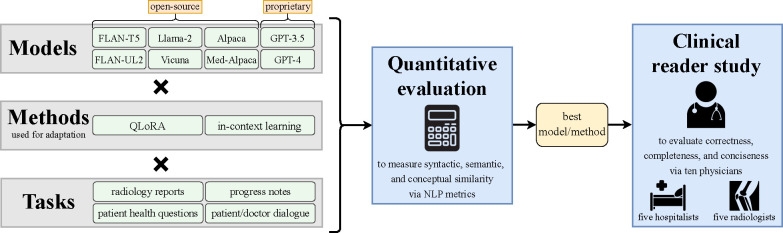
Overview. First we quantitatively evaluate each valid combination (×) of LLM and adaptation method across four distinct summarization tasks comprising six datasets. We then conduct a clinical reader study in which ten physicians compare summaries of the best model/method against those of a human expert.

**Table 1 |:** Model performance across different temperature values and expertise.

Parameter	Value	BLEU	ROUGE-L	BERTScore	MEDCON

Temperature	0.1	**4.9**	**28.1**	89.6	**28.2**
	0.5	4.9	27.1	**89.7**	27.5
	0.9	4.3	25.4	89.3	25.3

Expertise	None	10.4	34.3	90.2	30.7
	Medicine^1^	**11.1**	**35.5**	**90.5**	**35.5**
	Wizardry^2^	4.3	27.8	89.7	28.5

1: “You are an expert medical professional.”

2: “You are a mystical wizard in Middle Earth.”

**Table 2 |:** We quantitatively evaluate eight models, including state-of-the-art sequence-to-sequence and autoregressive models. Unless specified, models are open-source (vs. proprietary).

Model	Context	Parameters	Proprietary?	Seq2seq	Autoreg.

FLAN-T5	512	2.7B	-	✔	
FLAN-UL2	2,048	20B	-	✔	
Alpaca	2,048	7B	-	-	✔
Med-Alpaca	2,048	7B	-	-	✔
Vicuna	2,048	7B	-	-	✔
Llama-2	4,096	7B, 13B	-	-	✔
GPT-3.5	16,384	175B	✔	-	✔
GPT-4	32,768	unknown	✔	-	✔

**Table 3 |:** Description of four distinct summarization tasks comprising six open-source datasets with a wide range of token length and lexical variance, i.e. $\frac{\text{number}\;\text{of}\;\text{unique}\;\text{words}}{\text{number}\;\text{of}\;\text{total}\;\text{words}}$.

Task (Dataset)	Task description	Number of samples	Avg. number of tokens		Lexical variance
			Input	Output	

Radiol. reports (Open-i)	findings → impression	3.4K	52 ± 22	14 ± 12	0.11
Radiol. reports (MIMIC-CXR)	findings → impression	128K	75 ± 31	22 ± 17	0.08
Radiol. reports (MIMIC-III)	findings → impression	67K	160 ± 83	61 ± 45	0.09
Patient questions (MeQSum)	verbose → short question	1.2K	83 ± 67	14 ± 6	0.21
Progress notes (ProbSum)	notes → problem list	755	1,013 ± 299	23 ± 16	0.15
Dialogue (ACI-Bench)	dialogue → assessment	126	1,512 ± 467	211 ± 98	0.04

## Appendix

**Table A1 |:** Instructions for each of the four summarization tasks. For full prompt, see Figure 2.

Task	Instruction

Radiology reports	“Summarize the radiology report findings into an impression with minimal text.”
Patient questions	“Summarize the patient health query into one question of 15 words or less.”
Progress notes	“Based on the progress note, generate a list of 3-7 problems (a few words each) ranked in order of importance.”
Dialogue	“Summarize the patient/doctor dialogue into an assessment and plan.”

**Table A2 |:** Reader study results evaluating completeness, correctness, conciseness (columns) across individual readers. Scores are on the range [−10, 10], where positive scores denote GPT-4 is preferred to the human reference. Intensity of highlight colors blue (GPT-4 wins) or red (human wins) correspond to the score. See Figure 4 for further details and p-values.

Task	Reader	Completeness	Correctness	Conciseness

Radiology reports	1	3.5 ± 5.6	1.7 ± 3.6	1.2 ± 4.8
	2	3.6 ± 6.6	2.5 ± 4.7	−0.3 ± 5.4
	3	0.8 ± 2.9	0.6 ± 3.2	−1.7 ± 3.0
	4	4.7 ± 4.7	2.9 ± 3.9	1.2 ± 3.8
	5	1.4 ± 4.0	0.6 ± 2.2	−0.6 ± 3.4
	Pooled	2.8 ± 5.1	1.7 ± 3.7	0.0 ± 4.3

Patient questions	1	1.9 ± 7.1	0.8 ± 3.3	0.3 ± 3.0
	2	1.0 ± 5.6	−0.1 ± 3.6	0.1 ± 3.6
	3	2.3 ± 7.2	2.0 ± 5.3	2.2 ± 5.9
	4	1.9 ± 6.7	0.0 ± 0.0	0.0 ± 0.0
	5	0.9 ± 5.7	0.4 ± 3.6	0.4 ± 3.6
	Pooled	1.6 ± 6.5	0.6 ± 3.7	0.6 ± 3.9

Progress notes	1	3.4 ± 7.5	0.5 ± 2.5	0.1 ± 4.5
	2	2.3 ± 6.5	0.6 ± 4.4	0.4 ± 4.2
	3	2.7 ± 6.3	1.0 ± 4.4	0.9 ± 3.7
	4	2.5 ± 7.2	0.5 ± 6.8	1.7 ± 6.9
	5	2.0 ± 6.8	−0.8 ± 4.5	−0.1 ± 1.2
	Pooled	2.6 ± 6.9	0.4 ± 4.8	0.6 ± 4.5

**Table A3 |:** Intra-reader correlation values on a range of [−1, 1] where −1, 0, and +1 correspond to negative, no, and positive correlations, respectively.

Task	Completeness	Correctness	Conciseness

Radiology reports	0.45	0.58	0.48
Patient questions	0.67	0.31	0.21
Progress notes	0.77	0.74	0.42

Overall	0.63	0.56	0.38
